# Selection of Lipases for the Synthesis of Biodiesel from Jatropha Oil and the Potential of Microwave Irradiation to Enhance the Reaction Rate

**DOI:** 10.1155/2016/1404567

**Published:** 2016-10-27

**Authors:** Livia T. A. Souza, Adriano A. Mendes, Heizir F. de Castro

**Affiliations:** ^1^Department of Chemical Engineering, Engineering School of Lorena, University of São Paulo, 12602-810 Lorena, SP, Brazil; ^2^Institute of Chemistry, Federal University of Alfenas, 37130-001 Alfenas, MG, Brazil

## Abstract

The present study deals with the enzymatic synthesis of biodiesel by transesterification of Jatropha oil (*Jatropha curcas* L.) with ethanol in a solvent-free system. Seven commercial lipase preparations immobilized by covalent attachment on epoxy-polysiloxane-polyvinyl alcohol composite (epoxy-SiO_2_-PVA) were tested as biocatalysts. Among them, immobilized lipases from* Pseudomonas fluorescens *(lipase AK) and* Burkholderia cepacia* (lipase PS) were the most active biocatalysts in biodiesel synthesis, reaching ethyl ester yields (FAEE) of 91.1 and 98.3% at 72 h of reaction, respectively. The latter biocatalyst exhibited similar performance compared to Novozym® 435. Purified biodiesel was characterized by different techniques. Transesterification reaction carried out under microwave irradiation exhibited higher yield and productivity than conventional heating. The operational stability of immobilized lipase PS was determined in repeated batch runs under conventional and microwave heating systems, revealing half-life times of 430.4 h and 23.5 h, respectively.

## 1. Introduction

Biodiesel, defined as monoalkyl esters of fatty acids derived from several oils and fats, has gained attention as a source of alternative fuel due to their important features such as biodegradability, nontoxicity, low emission of carbon monoxide, particulate matter, and unburned hydrocarbons [1, 2]. The global production of biodiesel was worth nearly 22.5 billion liters in 2012 [3], with a projection around 41.9 billion liters in 2020 [4]. Biodiesel has been broadly produced using edible oils as feedstocks such as those from rapeseed, soybean, sunflower, and palm, thus leading to a conflict with food supply [1]. Several plant oils, animal fats, microalgal oils, and waste oils (animal rendering, fish processing waste, and cooking oils) have been proposed as potential feedstocks for biodiesel production [1, 2, 5]. On an industrial scale, biodiesel has been preferentially synthesized from methanol [6]. In the last years, the application of ethanol as a sustainable reactant has gained significant attention in industrial processes due to its high production and low cost in Brazil [6, 7].

Recent studies have focused on the application of nonedible oils for biodiesel production such as wastes and several plant oils, including from* Jatropha curcas* L. Its oil has been considered one of the most promising feedstocks for biodiesel production due to its high oil content, ranging from 30% to 50% based on seed dry mass [8]. Moreover, Jatropha trees can grow on wasteland with minimum water and fertilizer demand [9]. However, its application as feedstock presents some disadvantages such as high toxicity of their seeds and leaves to human beings and animals because the presence of toxic compounds such as curcin and phorbol esters limits its application in food industries and competition with food production for land use and the plant does not produce high amounts of seed oils unless it is well cultivated [10, 11].

The traditional route of biodiesel synthesis is the transesterification of oils and fats from several sources with short-chain alcohols such as methanol and ethanol [12]. For industrial biodiesel, homogeneous alkali catalysts such as potassium and sodium hydroxides, as well as potassium and sodium alkoxides, have been preferentially used [12, 13]. The application of acid catalysts has also been reported, but this process is slower than the alkali catalyzed process [2]. Acid catalyzed reactions are more feasible for the esterification and transesterification of oils and fats containing high free fatty acid content [2, 13]. However, there are major drawbacks of conventional chemical processes, as several problems during the steps of removal of catalyst from the product and the wastewater treatment, and excessive consumption of energy [2, 14]. In recent years, heterogeneous catalysts, including immobilized lipases, have gained much attention because they can be easily separated from the system at the end of the reaction and may also be reused. Besides, the use of these catalysts does not produce soaps by free fatty acid neutralization or triacylglycerol saponification [2, 13–15].

Lipases (triacylglycerol acyl hydrolases EC 3.1.1.3) are hydrolases that catalyze the hydrolysis of triacylglycerols into free fatty acids and glycerol, but they can also catalyze esterification, transesterification, and interesterification in nonaqueous media [16, 17]. The limitations of the industrial application of lipases have mainly been due to their high cost. Immobilization facilitates the separation of products and provides more flexibility with enzyme/substrate contact using various reactor configurations [16]. Moreover, immobilization on solid supports may improve enzyme features, from stability to selectivity. The immobilization of lipases by several techniques on solid supports is a well-documented technology that allows application of these enzymes in several industrial processes, including biodiesel synthesis [15–17]. Lipases have been immobilized using different protocols as physical adsorption on hydrophobic and ionic exchange resins, covalent attachment on highly activated supports, and encapsulation in organic matrices [16, 18–20]. Covalent attachment of nucleophilic groups of lipases (N-terminal and hydroxyl groups) on highly activated supports (e.g., epoxy or aldehyde groups on the support surface introduced after the activation step) may promote the stabilization of their three-dimensional structures, thus reducing possible conformational changes induced by any distorting agents such as solvents and temperature [18, 21–24]. Previous studies carried out in our lab have reported the preparation of robust biocatalysts prepared by covalent attachment of several lipases on activated hybrid support SiO_2_-PVA for further application in biotransformation reactions [21, 22, 25].

The aim of this study was the selection of immobilized lipases from several sources via covalent attachment on epoxy-SiO_2_-PVA particles to catalyze biodiesel synthesis (ethyl esters) by transesterification reaction of Jatropha oil in a solvent-free system. The results were compared with commercially available Novozym 435, the most used immobilized lipase in biotransformation reactions which is prepared by immobilizing CALB via physical adsorption on macroporous poly(methyl methacrylate-*co*-divinylbenzene) particles (Lewatit VP OC 1600) [26]. The most active biocatalyst was then selected for subsequent studies concerning the characterization of purified product by several techniques. Moreover, experiments were also carried out to determine the feasibility of using nonconventional heating systems such as microwave irradiation. Microwave, a nonionizing radiation has been explored in recent years as an alternative heating system in many areas, including the enzymatic biodiesel synthesis [27–29]. Microwave heating involves directed absorption of energy by functional groups that bear ionic conductivity or a dipole rotational effect, and this energy is then released into the surrounding solution [28]. In this study, seven biocatalysts were prepared via covalent attachment on epoxy-SiO_2_-PVA. Their catalytic properties were determined in hydrolysis and transesterification reactions. The results were compared with a commercial biocatalyst, Novozym 435. The application of these different biocatalysts in biodiesel production by transesterification with ethanol in a solvent-free system has not been reported in the literature yet.

## 2. Materials and methods

### 2.1. Materials

Lipase preparations from* Aspergillus niger *(lipase A),* Mucor javanicus* (lipase M),* Burkholderia cepacia *(lipase PS), and* Pseudomonas fluorescens *(lipase AK) were acquired from Amano Enzyme Inc. (Nagoya, Japan). Lipases from* Rhizopus oryzae *(lipase L036P) from (Cardiff, England) and* Rhizopus oryzae* (Piccantase R8000®) were purchased from DSM Food Specialities (Delft, The Netherlands). Porcine pancreatic lipase (type II) and Novozym 435 were purchased from Sigma-Aldrich Co. (St. Louis, MO, USA). All lipase preparations were used as received without further purification. Tetraethoxysilane was acquired from Aldrich Chemical Co. (Milwaukee, WI, USA). Anhydrous ethanol (minimum 99% wt) was purchased from Cromoline (São Paulo, SP, Brazil). Epichlorohydrin, hydrochloric acid (minimum 36% wt), polyvinyl alcohol (molecular mass 72,000 g/mol), and polyethylene glycol (molecular mass 1500 g/mol) were supplied by Reagen (Rio de Janeiro, RJ, Brazil). Olive oil (low acidity) from Carbonell (Spain) was purchased from a local market. Arabic gum was purchased from Synth® (São Paulo, SP, Brazil). Jatropha oil was kindly supplied by Instituto Agronômico do Paraná (IAPAR) (Londrina, PR, Brazil), and their main properties are given in Table 1. The fatty acid composition and physicochemical properties were determined according to AOCS (American Oil Chemists' Society) methods [31]. The organic solvents were of standard laboratory grade from Synth. All the other reagents were of analytical degree acquired from Synth and Vetec Química Ltda.

### 2.2. Support Synthesis and Lipase Immobilization Procedure

Epoxy-silica-PVA composite was prepared by the hydrolysis and polycondensation of tetraethoxysilane, following activation with epichlorohydrin according to the methodology previously described [22]. Before immobilization, the protein concentrations [32] and hydrolytic activities [33] of all lipase preparations were determined. The immobilization of the lipases was performed at a ratio of 1 : 4 gram of crude enzyme per gram of support using hexane as a dispersion medium in the presence of PEG-1500 (100 *μ*L/g of support). Lipase-support systems were kept in contact for 16 h at 4°C under static conditions and then the biocatalysts were filtered (nylon membrane 62HD from Scheiz Seidengaze-fabrik AG, Thal Schweiz, Switzerland) and thoroughly rinsed with hexane. The hydrolytic activity of lipase preparations was assayed by the olive oil emulsion method [33]. One international unit (IU) of enzyme activity was defined as the amount of enzyme that liberates 1 *μ*mol of free fatty acid per min under the assay conditions (37°C, pH 7.0). Specific activity was calculated after determining the activity of the immobilized enzyme (apparent hydrolytic activity) and comparing with the immobilized protein (IP) concentration. In this study, immobilization yield ≈100% for all immobilization procedures was observed.

### 2.3. Biodiesel Synthesis in Conventional Heating

The reactions were carried out in a jacketed cylindrical glass reactor (6 mm high × 4 mm internal diameter and 50 mL capacity, coupled with a reflux condenser) containing 15 g of substrate consisting of Jatropha oil to ethanol at molar ratio of 1 : 9 in a solvent-free system. The mixtures were incubated with the prepared biocatalysts at fixed proportion of 500 IU per gram of oil under agitation (200 rpm) at 45°C for a maximum period of 72 h. The reaction progress was monitored by taking samples at various time intervals and the ethyl esters formed were analyzed using a gas chromatography (Varian CG 3800, Inc., Palo Alto, CA, USA) with FID detection and a 5% DEGS CHR-WHP 80/100 mesh 6 ft 2.0 mm ID and 1/800 OD column (Restek Frankel Commerce of Analytic Instruments Ltd., SP, Brazil) following previous established conditions [21, 34].

### 2.4. Biodiesel Synthesis under Microwave Irradiation

The reactions were carried out in a microwave reactor (Model Discover, CEM Corporation) consisting of a cylindrical internal chamber with 75 mm diameter and 100 mm height. The temperature of the reaction was monitored by an infrared sensor located in the lower part of the chamber. ChemDriver software was used to record data for each run, including variation in the microwave flux and temperature evolution. The working volume was a 100 mL glass reactor containing 12 g of substrate consisting of Jatropha oil to ethanol at molar ratio oil to ethanol of 1 : 9 in a solvent-free system. The reactor was coupled with a reflux condenser to avoid ethanol loss. The mixture was also incubated with immobilized lipase at proportion of 500 IU per gram of oil at 45°C for a maximum period of 24 h. Ethyl esters formed were determined by GC analysis [34], and the FAEE yield (%) was defined as the ratio between the produced and theoretical esters concentrations. The productivity (mg/g·h) was calculated by dividing the ethyl esters concentration at a given time *t*.

### 2.5. Operational Stability Tests

The operational stability of the selected biocatalyst was carried out in both conventional heating and microwave irradiation systems by measuring the ethyl ester synthesis by GC analysis at the end of each reaction in the batch systems (runs 1−7), taking the original activity as 100%. The recovered immobilized lipase was then washed with* tert*-butanol to remove any substrate or product eventually retained in the biocatalyst microenvironment as glycerol. The inactivation constant (*k*_*d*_) and half-life (*t*_1/2_) for the immobilized lipase were calculated according to (1), as follows [22]:

(1) ln At=ln A0−kd·t,t1/2=ln 2kd,

where *A*_0_ is the initial activity of the immobilized lipase and *A*_*t*_ is the final activity after each run.

### 2.6. Purification of Ethyl Esters and Characterization

At the end of the reactions, the biocatalysts were recovered by centrifugation (4000 ×g) at room temperature for 10 min. The liquid phase was transferred into decanting funnel in which the same amount of distilled water was added. Then, vigorous stirring was carried out and the mixture was allowed to stand for 6 h, for phase separation. This procedure was performed three times, in sequence. The superior phase, consisting of fatty acid ethyl esters (biodiesel), was evaporated in a rotatory evaporator. Subsequently, the solution was dried sodium sulfate and the lower phase, consisting of glycerol and wastewater, was discharged.

The ^1^H NMR spectroscopy was carried out on a Varian Mercury spectrometer operating at 300 MHz. The samples (around 120 mg) were dissolved in 0.6 mL of CDCl_3_ that contained 0.03% tetramethylsilane, and the resulting solutions were placed in a 5 mm diameter NMR tube. The kinematic viscosity of purified ethyl esters was determined by Brookfield Viscometers model LVDVII (Brookfield Viscometers Ltd., UK) using the cone CP 42. Thermogravimetry (TGA) and differential thermogravimetry (DTG) curves for Jatropha oil and ethyl esters (biodiesel) were recorded using a thermal balance (Shimadzu TGA-50, Thermogravimetric Analyzer). A dynamic method was used, at a heating rate of 10°C/min. The initial sample mass was 10.00 ± 0.5 mg, in both inert (nitrogen) and oxidative (synthetic air) atmospheres with a flow rate of 50 mL/min, in the 25–1000°C temperature range [35]. FT-IR analysis was used in order to confirm the purity of biodiesel sample. The IR spectrum was recorded in a Perkin Elmer Spectrum GX spectrometer using KBr pellets in the range of 4000–400 cm^−1^.

### 2.7. Biodiesel Properties Based on the Jatropha Oil Fatty Acids Profile

In this study, biodiesel properties based on fatty acids profile from Jatropha oil were estimated using the software “Biodiesel Analyzer version 1.1” (available on http://www.brteam.ir/biodieselanalyzer) [30].

## 3. Results and Discussion

### 3.1. Catalytic Properties of the Lipase Preparations

 Seven commercial lipase preparations were used as biocatalysts and their catalytic properties were determined in terms of protein concentration, hydrolytic activity, and specific activity (ratio between the hydrolytic activity and protein concentration). According to Table 2, Piccantase R8000, porcine pancreatic, and lipase M presented the highest protein concentration, followed by lipase A and lipase L036P. Among them, lipase PS and lipase AK exhibited the lowest protein concentrations, 13.0 ± 0.3 and 14.2 ± 0.4 mg/g of crude extract, respectively.

Lipase L036P presented the maximum hydrolytic activity (45112.4 ± 907.7 IU/g of crude extract), followed by lipase M (40875.4 ± 1220.8 IU/g of crude extract). The hydrolytic activities of lipase PS and lipase AK were, respectively, 30422.8 ± 1044.5 and 29878.5 ± 890.6 IU/g of crude extract. Among the tested biocatalysts, lipase A, porcine pancreatic, and Piccantase R8000 were the less active biocatalysts in the hydrolysis of olive oil emulsion. Although both lipase L036P and Piccantase R8000 are lipase preparations from* R. oryzae*, the latter presented higher protein concentration, as above described, and hydrolytic activity around 3.26-fold lower than lipase L036P. These different features could be attributed to different cultivation conditions and inducers used in the production of each lipase preparation that strongly influence their catalytic properties, including the production of isoenzymes and glycosylation degree [36]. Moreover, different additives and stabilizing agents present in their formulations such as salts, polyols, or sugars and purification degree could also influence their catalytic properties [37].

The specific activity for each lipase preparation (crude extract) was also determined, as shown in Table 2. This parameter is more interesting due to the low purity of commercial lipase preparations and the specific activity allows a real and effective comparison among enzymatic activities towards a given substrate [38]. Although lipase PS and lipase AK have presented the lowest protein concentrations, these two lipase preparations exhibited higher specific activity values than those of the other preparations, 5416.7 ± 102.6 and 2133.9 ± 87.5 IU/mg of protein, respectively. Specific activity values for lipase A, lipase M, lipase L036P, Piccantase R8000, and porcine pancreatic varied from 104.2 ± 7.3 to 484.1 ± 21.9 IU/mg of protein. The results showed that specific activity follows the series lipase PS > lipase AK > lipase L036P > lipase M > lipase A > porcine pancreatic > Piccantase R8000.

The hydrolytic and specific activity values of the prepared biocatalysts were also determined (Table 2). The hydrolytic activity of the immobilized lipases ranged from 749.1 ± 51.7 IU/g of support (immobilized Piccantase R8000) to 2925.4 ± 100.8 IU/g of support (immobilized lipase L036P) and shows different behavior of each lipase preparation for the support epoxy-SiO_2_-PVA. The immobilization of lipase PS and lipase AK yielded also biocatalysts with high hydrolytic activity, 1980.6 ± 88.4 IU/g and 1860.3 ± 94.5 IU/g, respectively. These biocatalysts were strongly more active than those ones prepared by immobilizing lipase M, porcine pancreatic, lipase A, and Piccantase R8000.

Specific activity values for all prepared biocatalysts were lower than the ones previously determined for crude lipase preparations, as shown in Table 2 (between 31.4 ± 0.9 and 609.4 ± 27.2 IU/mg_IP_). These results suggest that upon immobilization the support surface promoted a strong distortion on the three-dimensional structure of lipases or bad orientation on the support surface that restricted the access of olive oil molecules to their active sites. These results could be also attributed to the different additives and stabilizing agents present in each formulation. These additives have been used to protect the enzyme from external denaturing agents by providing additional sites for hydrogen bonding with the enzyme surface that decreases dehydration and distortion of its three-dimensional structure [39]. However, these additives can interact with nucleophilic groups in the enzyme structure (e.g., –NH_2_ and –OH) that influence the orientation of enzyme molecules on the support surface [40, 41]. Similar results were reported in previous studies carried out in our lab concerning the immobilization of* Thermomyces lanuginosus* lipase from different commercial formulations supplied by Novozymes (Lipolase® and Lipex® 100 L) by different protocols on activated organic supports (glyoxyl-agarose and epoxy-chitosan/alginate beads) [23, 24].

### 3.2. Selection of Biocatalysts in Ethyl Esters Synthesis

Lipases display high specificity towards different length and type of fatty acids of triacylglycerol molecules (acyl donor) and the length of alcohol (acyl acceptor) [42]. Thus, lipases should be nonstereo specific, so that all tri-, di-, and monoacylglycerols can be converted to the correspondent monoalkyl esters (biodiesel). At the same time, they should also catalyze the esterification of free fatty acids [43].

The immobilized lipases were used in biodiesel synthesis from Jatropha oil in order to select the most active biocatalysts. In this set of experiments, transesterification reactions were performed at molar ratio oil to ethanol of 1 : 9 for 72 h reaction under continuous agitation (200 rpm) at 45°C using 500 IU per gram of oil.

According to results summarized in Table 3, ethyl esters from palmitic (C16:0), stearic (C18:0), oleic (C18:1), and linoleic (C18:2) acids were the main esters produced in the reaction. Among the tested biocatalysts, immobilized lipase M, lipase A, and Piccantase R8000 displayed the lowest catalytic activities, rendering maximum FAEE yield of 1.8, 2.3, and 4.1%, respectively (Table 3). Thus, kinematic viscosity values for these samples were almost identical to the crude oil. The reaction catalyzed by immobilized lipase L036P and porcine pancreatic attained similar FAEE yields (around 9%) and kinematic viscosities (25.9–27.5 mm^2^/s). However, these biocatalysts displayed different specificities because porcine pancreatic exhibited higher affinity towards palmitic acid (C16:0), while lipase L036P showed higher affinity for stearic acid (C18:0).

The most active biocatalysts were those ones prepared by immobilizing lipase AK and lipase PS that attained FAEE yields of 91.1 and 98.3%, corresponding to 59.3 and 63.9% wt of ethyl esters, respectively.

**Figure 1:**
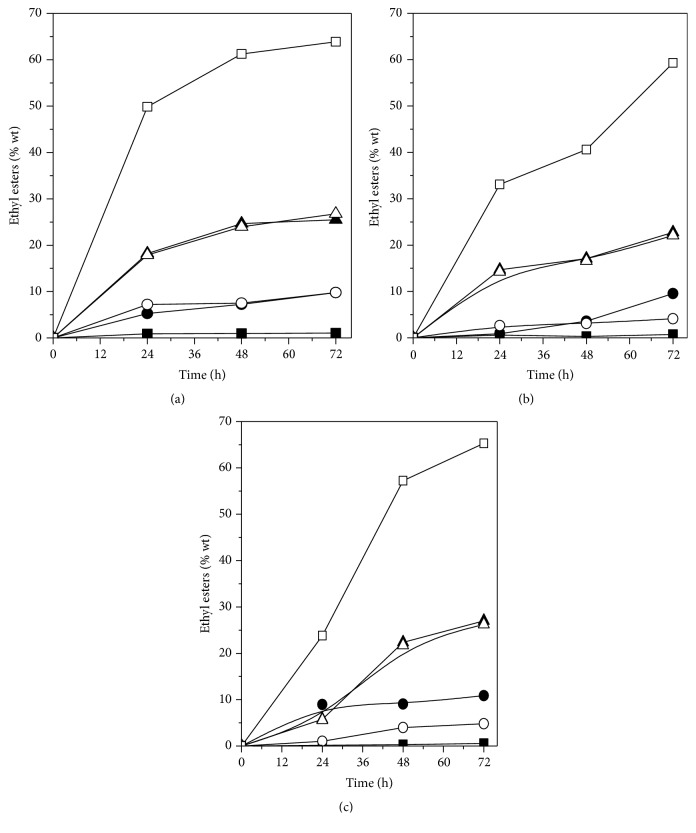
Profile for alkyl esters formation in the transesterification reaction of Jatropha oil catalyzed by immobilized lipase PS (a), lipase AK (b), and Novozym 435 (c). Symbols (-■- C14:0, -●- C16:0, -○- C18:0; -▲- C18:1, -Δ- C18:2, -□- total).

A comparison between the reaction performances by the two most active biocatalysts (immobilized lipases AK and PS) and Novozym 435 is displayed in Figures 1(a)–1(c) in terms of ethyl esters concentration (% wt) formed as a function of time. Maximum ethyl ester concentration of all lipases was reached at 72 h of reaction. However, immobilized lipase PS (Figure 1(a)) was slightly more active than immobilized lipase AK (Figure 1(b)) and presented similar performance compared to the commercial biocatalyst (Novozym 435) (Figure 1(c)). These results could be attributed to good diffusion of substrate molecules from the reaction mixture to the microenvironment of the prepared biocatalyst with lipase PS. Moreover, the kinematic viscosity for the purified samples from the reaction catalyzed by immobilized lipase PS and Novozym 435 is in agreement with specifications recommended by the ASTM D6751 for biodiesel (methyl and ethyl esters), which states that the kinematic viscosity of biodiesel (B100) must be in the range between 1.9 and 6.0 mm^2^/s. On the other hand, the kinematic viscosity of ethyl esters synthetized using immobilized Lipase AK was 6.72 mm^2^/s due to incomplete conversion of the oil into ethyl esters (FAEE yield of 91.1%).

**Figure 2:**
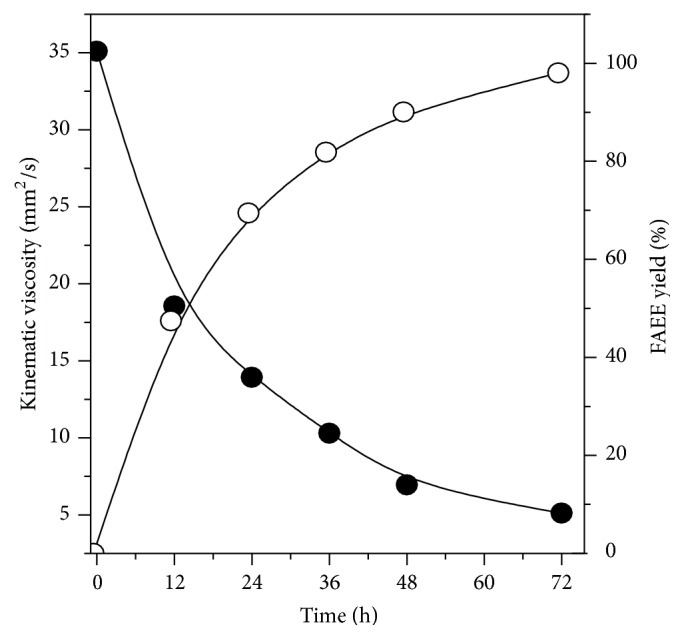
Relationship between transesterification yield (open circle) and kinematic viscosity (closed circle) as a function of the reaction time.

Viscosity is an important property of biodiesel since it affects the fluidity of the fuel and the operation of fuel injection equipment. High viscosity leads to poor atomization of the fuel spray and less accurate operation of fuel injectors [44].  Figure 2 shows the correlation between the FAEE yield and the kinematic viscosity reduction as a function of time for the reaction catalyzed by the immobilized Lipase PS, the most active prepared biocatalyst in this study. As can be observed, consistent reduction of the kinematic viscosity value from 35.4 to 5.1 mm^2^/s was attained as the FAEE yield increased, which is in accordance with specifications recommended by the ASTM for biodiesel (methyl and ethyl esters) (method ASTM D6751). Similar behavior was described in previous studies for the enzymatic synthesis of ethyl esters from several feedstocks [21, 45].

Based on these results, the characterization of purified ethyl esters (biodiesel) using different methods was carried out. Comparative study of ester synthesis in a conventional and microwave heating in both single and successive batch runs was also carried out using immobilized lipase PS as biocatalyst.

### 3.3. Characterization of the Purified Ethyl Esters by Several Techniques

**Figure 3:**
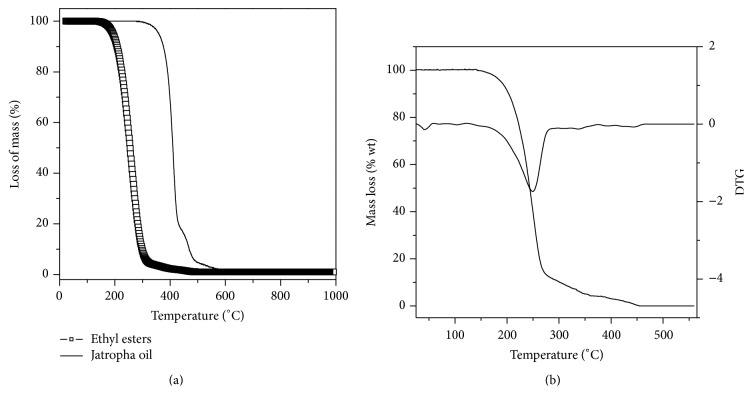
Thermogravimetric (TG) curves for Jatropha oil and biodiesel sample in nitrogen atmosphere (a) and TG/DTG curves for biodiesel sample in air atmosphere (b).

Thermogravimetric analysis was carried out to compare the thermal stability of Jatropha oil and ethyl esters (biodiesel).  Figure 3(a) shows the TG curves in inert atmosphere for oil and biodiesel obtained from the transesterification reaction catalyzed by immobilized lipase PS. Jatropha oil was thermally stable up to 321°C, thus showing its high thermal stability. TG curve showed two stages of thermal degradation between 321–441.7°C and 441.7–499.6°C, with 81.6 and 18.4% of mass loss, respectively, corresponding with the volatilization of fatty acids contained in Jatropha oil. On the other hand, biodiesel showed just one step of thermal degradation between 128 and 290°C with higher decomposition rate at 258°C. The loss of mass in the order of 98% was also attributed to volatilization of ethyl esters.

Thermogravimetric analysis was also performed to evaluate the oxidative stability of biodiesel from Jatropha oil since poor oxidative stability is one of the major issues that limit the use of biodiesel as a fuel in compression ignition engines [46].  Figure 3(b) shows the TG/DTG curves obtained for the biodiesel sample in an oxidative atmosphere. As can be observed, the mass of the biodiesel starts to decrease at approximately 140°C and it continues until all the ethyl esters present in the sample were vaporized. The relative mass lost was around 95%. Biodiesel from Jatropha oil had lower oxidative stability than those ones from beef tallow (*T*_initial_ = 187°C), Babassu oil (*T*_initial_ = 154°C), and palm oil (*T*_initial_ = 144°C) [47]. This result was expected due to its higher percentage of linoleic acid (see Table 1) that is prone to oxidative degradation. Similar behavior was reported for other polyunsaturated biodiesel from safflower, soybean, linseed, and sunflower oils [48]. Oxidation stability can be improved by antioxidant additives or blending with more oxidatively stable biodiesel [48].

**Figure 4:**
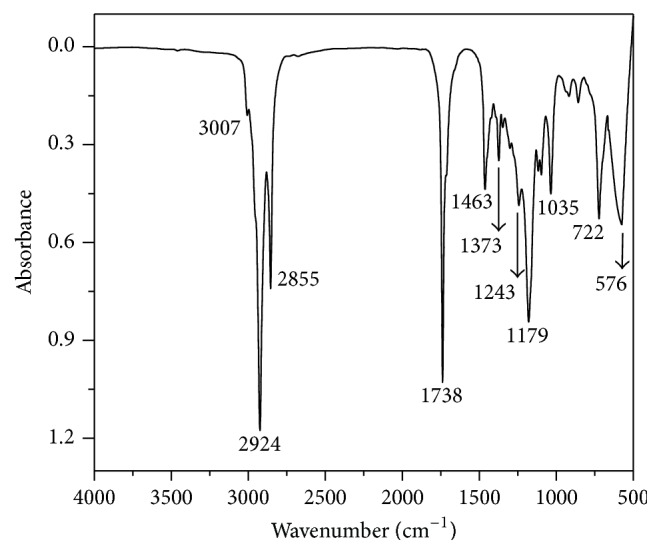
FT-IR spectrum for purified biodiesel from the transesterification reaction catalyzed by immobilized lipase PS.

The quality of the purified biodiesel was confirmed by infrared spectroscopy analysis (Figure 4). The infrared spectrum shows the ester C=O axial deformation at 1738 cm^−1^, as well as two bands related to the ester C–O axial deformation at 1243 and 1179 cm^−1^ [49]. The bands between 2924 and 2855 cm^−1^ correspond to the vibration of symmetrical and asymmetrical stretching of C–H methyl and methylene groups. A weak signal at 3007 cm^−1^ is due to the olefinic group (=CH–) which indicates the presence of unsaturated fatty acids in ethyl esters. The spectrum shows that the produced biodiesel did not display the characteristic band at around 3300 cm^−1^related to the primary alcohol O–H axial deformation. These results suggest absence of residual glycerol or ethanol molecules, thus showing that the step of biodiesel purification was very efficient and simple.

**Figure 5:**
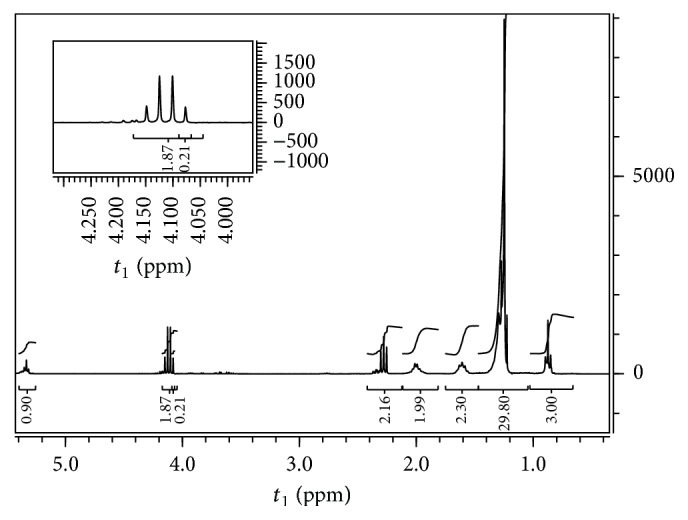
^1^H NMR spectrum for the purified biodiesel from the transesterification reaction catalyzed by immobilized lipase PS.

The use of NMR analysis could not only confirm the biodiesel quality but also allowed determining the conversion of vegetable oil into esters. It is possible to verify in Figure 5 the appearance of the signal a quartet at 4.1 ppm, referring to the protons of the ethylene group from the alcohol portion of ester [CH_3_–CH_2_–O–(C=O)–R] and the absence of signals corresponding to the hydrogen atoms of the CH_2_ group of the glycerol at 4.2 ppm [50]. With these results, the equation validated by Paiva et al. [51] was tested for the operating conditions of this study, obtaining a correlation coefficient of 0.994 and 98.5% conversion of Jatropha oil into ethyl esters. From these data, it was possible to demonstrate the efficiency of the enzymatic route for the synthesis of biodiesel from Jatropha oil, as well as the quality of the product obtained as fuel in terms of thermal stability, purity, and fluidity.

In addition, properties such as cetane number (CN), cold filter plugging point (CFPP), cloud point (CP), and oxidation stability (OS) are parameters widely used to characterize biodiesel and can be predicted based on fatty acids profile [30], as displayed in Table 4.

The CN is a measurement of the combustion quality during ignition of biodiesel and depends on the distribution of saturated and unsaturated fatty acids or esters. The ignition quality is associated with the interval of ignition. A fuel with high CN number has a short ignition interval and starts to burn shortly after it is injected into an engine. In this study, CN value was estimated at 53.0. This value is in compliance with the standards for biodiesel (ASTM D6751), which recommend a minimum CN of 47 [52]. The long chain saturated factor is used to calculate the cold filter plugging point (CFPP), which is based on the amount of long chain saturated fatty acids (from C16:0) in the oil. The CFPP value is related to the minimum temperature at which the biodiesel can generate clogging and problems in the motor. The biodiesel provided a CFPP value of 9.28°C. This value is lower compared with methyl esters (biodiesel) from palm and peanut oils, whose CFPP values are 10 and 17°C, respectively [52]. Concerning the oxidative stability, the predicted value (5.87 h) is outside the minimum required by the standard requirements (6.0 h at 110°C) though it has better stability than soybean biodiesel since the content of polyunsaturated ethyl esters (C18:2) is balanced with similar monounsaturated content (18:1) which is considered to have lower suitability to be degraded. From the comparison standpoint, our results had some characteristics similar to biodiesel samples obtained from other feedstocks [52]. In addition, the results described here are in close agreement with those reported by Giakoumis [48], in which a detailed statistical investigation was conducted to assess the average values of all properties of the most investigated biodiesel, including biodiesel from Jatropha oil.

### 3.4. Microwave Irradiation versus Conventional Heating for Ethyl Esters Synthesis

**Figure 6:**
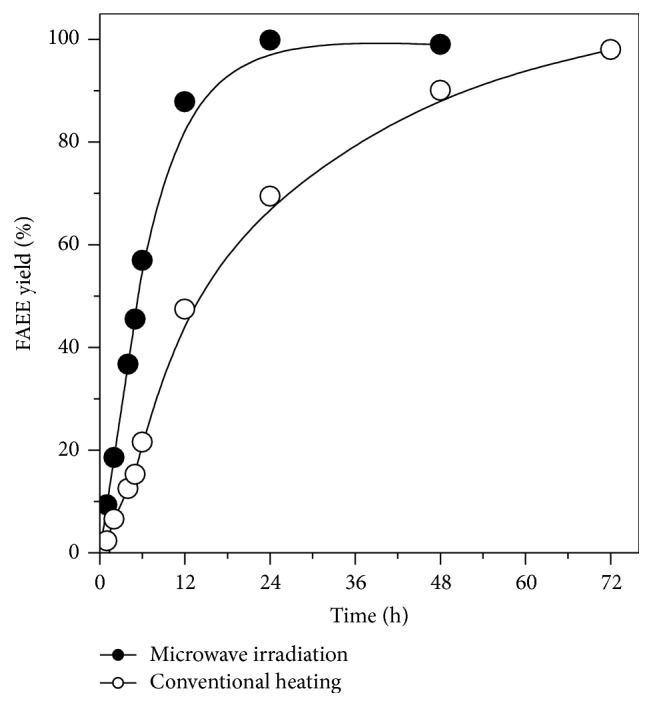
Comparison of ethyl esters yields under conventional heating (open circle) and microwave irradiation (closed circle) using immobilized lipase PS as biocatalyst.

In this study, transesterification of Jatropha oil with ethanol catalyzed by immobilized lipase PS under conventional heating and microwave irradiation conditions was carried out in order to evaluate the effect of the heating form on the ester synthesis.  Figure 6 shows the progress of the transesterification reaction in terms of FAEE yield (%) for the two forms of heating. Under conventional heating, a full conversion into ethyl esters was achieved at 72 h (productivity of 45 mg biodiesel/g·h), while the reaction reached equilibrium in 24 h using microwave heating with the same yield, corresponding to productivity of 121.4 mg biodiesel/g·h. This indicates that to achieve the same yield of ethyl esters yield percentage, shorter reaction time was needed under microwave irradiation compared to the conventional heating. Moreover, the reaction rate was improved up to 3-fold in the first 4 h of reaction compared to conventional heating.

Yadav and Lathi [53] also observed an increase of initial activity for the enzymatic transesterification of methyl acetoacetate with various alcohols in the 2.2–4.6-fold range for microwave irradiated reaction over conventional reactions. In another study, the enzymatic synthesis of isoamyl myristate by esterification reaction in a solvent-free system catalyzed by Novozym 435 was also improved by microwave irradiation [54]. The productivity of xylitol monoesters synthesis catalyzed by immobilized lipase from* Penicillium camemberti *was higher under nonconventional heating than conventional one, 5.3 mmol/h and 2.2 mmol/h, respectively [29].

It is known that microwave heating involves directed absorption of energy by functional groups that bear ionic conductivity or a dipole rotational effect, and this energy was then released into the surrounding solution [55]. In the reaction medium, ethanol is a good microwave radiation absorption material [28], which would destroy the two-tier structure of the interface between ethanol and oil, making the functional groups much more highly reactive [56]. Microwave irradiation can also induce conformation changes in the lipase structure that facilitates the access of substrate molecules to the active sites and, consequently, the enzyme behaves slightly differently and may become more active [27].

### 3.5. Biocatalyst Reuse under Successive Batch Runs

**Figure 7:**
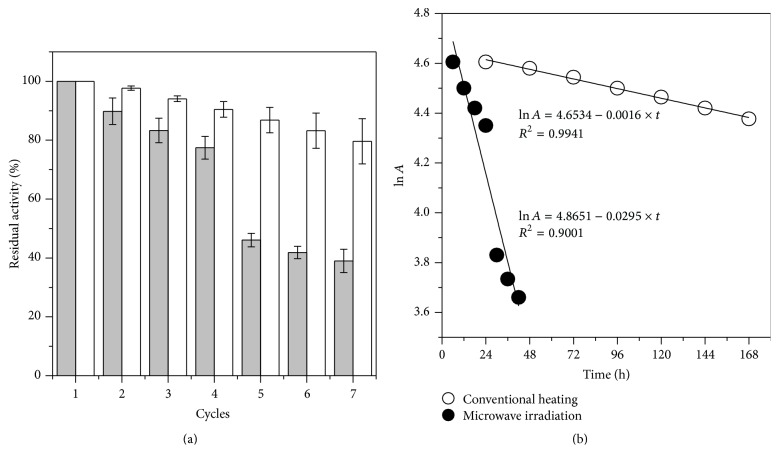
Operational stability of immobilized lipase PS after consecutive batch runs under conventional heating (white bars) and microwave irradiation (grey bars) (a) and plot of ln *A* versus reaction time for the determination of inactivation constant and half-life (b).

The operational stability of the selected biocatalyst (immobilized lipase PS) was determined in consecutive transesterification batch runs carried out under conventional heating (24 h at 45°C) and microwave irradiations (6 h at 45°C). Transesterification activity was determined by the formation of ethyl esters at the end of each recycle process as shown in Figure 7(a), taking the activity attained in the first run as 100%.

In both heating systems the production of esters was gradually decreased due to the loss of enzyme during filtration and drying (since no makeup quantities of enzyme were added), another possibility may be related to insufficient washing of the biocatalyst between the batches to remove the glycerol formed as byproduct which is adsorbed on the biocatalyst surface. However, higher inactivation rate under microwave irradiation was observed. The half-life (*t*_1/2_) and inactivation constant (*k*_*d*_) of the biocatalyst were determined by applying the first-order decay model (1), as shown in Figure 7(b). According to the results, *t*_1/2_ of immobilized lipase PS after successive runs of reaction under conventional heating (*k*_*d*_ = 0.0016 h^−1^) and microwave irradiation (*k*_*d*_ = 0.0295 h^−1^) was 430.4 h and 23.5 h, respectively.

This result was contradictory with the good prospects of enzymatic synthesis of esters of industrial interest catalyzed by Novozym 435. This biocatalyst showed higher stability under microwave irradiation compared to conventional heating in the methyl ester production from soybean oil [56]. Yu et al. [55] also demonstrated good stability and the possibility of recycling of Novozym 435 during microwave-assisted resolution of (*R,S*)-2-octanol with vinyl acetate by enzymatic transesterification. A slight decrease (8%) in enzyme activity was observed under microwave irradiation after five reuses. However, only 70% of the original activity of the biocatalyst was recovered under conventional heating after five reuses.

Deleterious effects of microwave irradiation on the operational stability of immobilized enzymes are still scarce in the literature. Some insight into the reason for lower operational stability under microwave irradiation may be gained by considering the medium polarity. Microwave irradiation exerts a strong selective heating by highly polar compounds (e.g., short-chain alcohols) due to their low log *P* values and high dielectric constant that affect the dipole molecular rotation and ion induction of these compounds, thus improving the energy transfer in the reaction mixture [54, 57]. In this study, transesterification reaction was carried out using excess of ethanol (molar ratio oil to ethanol of 1 : 9). Thus, a strong energy transfer and heating of nonreacted ethanol molecules could increase the interaction with the essential hydration layer surrounding the enzyme structure, thus leading to possible distortion of its active conformation. Moreover, microwave irradiation could also accelerate desorption of noncovalent enzyme molecules from the support surface after successive cycles of reaction.

## 4. Conclusion

The immobilization of several lipases via covalent attachment on epoxy-SiO_2_-PVA composite allowed the preparation of biocatalysts with different activities in hydrolysis and transesterification reactions. Among them, immobilized lipase from* Rhizopus oryzae* (L036P) was the biocatalyst with the highest hydrolytic activity. On the other hand, immobilized lipase PS and lipase AK were the most active biocatalysts for biodiesel synthesis. The promising performance of immobilized lipase PS was confirmed by TGA, FT-IR, and ^1^H NMR analyses indicating the occurrence of the transesterification reaction, by the corresponding thermal degradation of ethyl esters and the presence of bands related to formation of ester bonds and high purity. Additional experiments were carried out under microwave irradiation and the results suggested that the microwave heating constitutes a potential procedure to enhance the reaction rate by reducing the total reaction time. However, the immobilized lipase showed lower half-life time when subjected to consecutive bath runs under microwave irradiation, probably, that under these conditions the enzyme desorption from the support surface was more intense than conventional heating. These results clearly show the potential application of the selected biocatalyst to synthesize biodiesel in a solvent-free system.

## Figures and Tables

**Table 1:** Properties and fatty acid composition of Jatropha oil.

Property		Value
Kinematic viscosity at 40°C (mm^2^/s)		35.4
Acidic value (mg KOH/g oil)		0.3
Saponification value (mg KOH/g oil)		141
Iodine value (g I_2_/100 g oil)		101
Fatty acids composition (% wt)		
Lauric	C12:0	0.05
Myristic	C14:0	0.07
Pentadecanoic acid	C15:0	0.02
Palmitic	C16:0	12.2
Palmitoleic	C16:1	0.82
Margaric	C17:0	0.09
*cis*-10-Heptadecenoic	C17:1	0.06
Stearic	C18:0	5.46
Oleic	C18:1	38.98
Linoleic	C18:2	36.83
Linolenic	C18:3	0.72
Arachidic	C20:0	0.26
Eicosenoic	C20:1	0.46
Behenic	C22:0	3.62
Lignoceric	C24:0	0.11
Saturated (% wt)		21.88
Monounsaturated (% wt)		40.32
Polyunsaturated (2,3) (% wt)		37.55

**Table 2:** Catalytic properties of the lipase preparations used in this study (crude extracts and immobilized on epoxy-SiO_2_-PVA).

Lipase source	Designation	Crude lipases			Immobilized lipases	
		Protein^a^ (mg/g)	Hydrolytic activity^b^ (IU/g)	Specific activity^c^ (IU/mg of protein)	Hydrolytic activity (IU/g of support)	Specific activity^d^ (IU/mg_IP_)
*Aspergillus niger*	Lipase A	96.3 ± 1.3	23750.9 ± 370.2	246.6 ± 15.4	756.8 ± 21.4	31.4 ± 0.9
*Mucor javanicus*	Lipase M	121.4 ± 3.7	40875.4 ± 1220.8	337.8 ± 18.3	1286.3 ± 77.3	42.5 ± 2.5
*Rhizopus oryzae*	Lipase L036P	93.5 ± 1.9	45112.4 ± 907.7	484.1 ± 21.9	2925.4 ± 100.8	125.0 ± 4.3
*Rhizopus oryzae*	Piccantase R8000	133.9 ± 2.4	13848.1 ± 268.7	104.2 ± 7.3	749.1 ± 51.7	22.4 ± 1.5
*Burkholderia cepacia*	Lipase PS	13.0 ± 0.3	30422.8 ± 1044.5	5416.7 ± 102.6	1980.6 ± 88.4	609.4 ± 27.2
*Pseudomonas fluorescens*	Lipase AK	14.2 ± 0.4	29878.5 ± 890.6	2133.9 ± 87.5	1860.3 ± 94.5	523.9 ± 26.7
Porcine pancreatic	Pancreatic	121.8 ± 2.1	19802.7 ± 371.4	162.3 ± 12.4	1059.8 ± 42.9	34.8 ± 1.4

^a^Protein concentration determined by the Bradford method.

^b^Hydrolytic activity measured on the olive oil emulsion hydrolysis (pH 7.0, 37°C).

^c^Calculated as the hydrolytic activity of the enzyme per milligram of protein.

^d^Calculated as the hydrolytic activity of the enzyme per milligram of immobilized protein (IP).

**Table 3:** Ethyl esters profile, FAEE yield, and kinematic viscosity for biodiesel synthesis from the transesterification reaction of Jatropha oil catalyzed by different biocatalysts.

Lipase source	Ethyl esters (% wt)						FAAE yield (%)	Kinematic viscosity (mm^2^/s)
	C14:0	C16:0	C18:0	C18:1	C18:2	Total		
Lipase A	0.1	0.9	0.1	0.1	0.1	1.5	2.3	33.0
Lipase M	0.5	0.2	0.1	0.2	0.2	1.2	1.8	27.3
Lipase L036P	0.1	0.5	0.4	2.4	2.3	5.7	8.7	25.9
Piccantase R8000	0.6	1.0	0.1	0.5	0.5	2.7	4.1	31.6
Lipase PS	1.0	5.3	5.4	25.5	26.8	63.9	98.3	5.1
Lipase AK	0.7	9.6	4.1	22.8	22.1	59.3	91.1	6.7
Porcine pancreatic	0	2.1	0.0	2.4	2.1	6.6	10.1	27.5
Novozym 435	0.2	8.1	4.8	25.0	26.3	64.4	98.9	5.4

**Table 4:** Jatropha biodiesel properties obtained in the transesterification reaction with ethanol catalyzed by immobilized lipase PS on epoxy-SiO_2_-PVA.

Biodiesel property	Value	
	Experimental	Predicted^*∗*^
Ester content (%)	98.3 ± 1.2	—
Density at 20°C (kg/m^3^)	883.0 ± 0.5	820
Kinematic viscosity at 40°C (mm^2^/s)	5.1 ± 0.5	1.27
Saponification value (SV) (mg KOH/g oil)		188.2
Iodine value (IV) (g I_2_/100 g oil)		99.4
Cetane number (CN)		53.0
Cold filter plugging point (CFPP) (°C)		9.28
Cloud point (CP) (°C)		1.32
Oxidation stability (OS) (h)		5.87
Higher heating value (HHV) (MJ/kg)		37.16

^*∗*^Calculated using the software Biodiesel Analyzer version 1.1 [30].
